# Quantitative digital *in situ *senescence-associated β-galactosidase assay

**DOI:** 10.1186/1471-2121-12-16

**Published:** 2011-04-15

**Authors:** Liran I Shlush, Shalev Itzkovitz, Ariel Cohen, Aviad Rutenberg, Ron Berkovitz, Shiran Yehezkel, Hofit Shahar, Sara Selig, Karl Skorecki

**Affiliations:** 1 Laboratory of Molecular Medicine, Rappaport Faculty of Medicine and Research Institute, Technion, Haifa 31096, Israel; 2 Israeli Naval Medical Institute, Haifa, Israel; 3 Rambam Health Care Center, POB 9602, Haifa 31096, Israel; 4 Department of Computer Science and Applied Mathematics, Weizmann Institute of Science, Rehovot 76100 Israel

## Abstract

**Background:**

Cellular senescence plays important roles in the aging process of complex organisms, in tumor suppression and in response to stress. Several markers can be used to identify senescent cells, of which the most widely used is the senescence-associated β-galactosidase (SABG) activity. The main advantage of SABG activity over other markers is the simplicity of the detection assay and the capacity to identify *in situ *a senescent cell in a heterogeneous cell population. Several approaches have been introduced to render the SABG assay quantitative. However none of these approaches to date has proven particularly amenable to quantitative analysis of SABG activity *in situ*. Furthermore the role of cellular senescence (CS) *in vivo *remains unclear mainly due to the ambiguity of current cellular markers in identifying CS of individual cells in tissues.

**Results:**

In the current study we applied a digital image analysis technique to the staining generated using the original SABG assay, and demonstrate that this analysis is highly reproducible and sensitive to subtle differences in staining intensities resulting from diverse cellular senescence pathways in culture. We have further validated our method on mouse kidney samples with and without diabetes mellitus, and show that a more accurate quantitative SABG activity with a wider range of values can be achieved at a pH lower than that used in the conventional SABG assay.

**Conclusions:**

We conclude that quantitative *in situ *SABG assay, is feasible and reproducible and that the pH at which the reaction is performed should be tailored and chosen, depending on the research question and experimental system of interest.

## Background

Cellular senescence (CS) is a term used to describe the process wherein somatic cells of complex eukaryotic organisms progressively lose replicative capacity. The relationship between CS and organismal aging is still unclear although recent studies in non-human and human primates have strongly implicated a correlation between organismal and cellular aging [1,2]. Overall, recent studies have suggested that CS is a final common pathway resulting from activation of the cellular DNA damage response (DDR) by various stressors that converge on the p53 and/or pRB pathways. Different DDR inducing stimuli can lead to various types of CS. Among those most thoroughly investigated is the activation of DDR by telomere attrition which leads to cell cycle arrest termed replicative senescence (RS) or telomere-initiated CS [3-5]. Other well studied forms of CS include oncogene-induced senescence [6-8], cell structure induced senescence related to dysfunctional Lamin A [9], and stress-induced premature senescence (SIPS), the latter most thoroughly studied in relation to oxidative stress [10-12]. These various triggers of CS might not necessarily be mutually exclusive. Furthermore, DDR might not be the exclusive mechanism for triggering CS as protein damage, epigenetic changes [13] and additional processes have also been implicated [5,14]. In complex long-lived organisms CS is considered to be a tumor suppressor mechanism similar to apoptosis and autophagy [15]. However, in contrast to apoptosis and autophagy, which are irreversible and lead to cell death, senescent cells maintain partial metabolic functionality without dividing, and have been shown to have the capacity to revert back to a proliferative state [14].

Several markers of senescence have been described [5]. Among others these include G1 cell cycle arrest detected by lack of DNA replication, cytological markers such as senescence-associated heterochromatin foci (SAHF), senescence-associated DNA-damage foci, as well as cell structure changes such as cell size and lysosomal β-galactosidase activity detected at pH 6.0 defined as senescence-associated β-galactosidase (SABG) activity [16,17]. Since first reported, SABG activity has been the most extensively utilized biomarker for CS both in *in situ *[16,18-20] and in *in vitro *studies (reviewed in [17]). In many studies the identification of cells as being senescent rests solely on the SABG assay. The popularity of this method can be attributed to its simplicity and apparent specificity for CS regardless of the initiating trigger, as well as the ability to visualize senescent cells in a heterogeneous population [17].

Despite the extensive utilization of the SABG assay for CS determination, the origin of SABG activity and its role in CS were unknown for several years following its initial description. A number of studies have proposed that lysosomal β-galactosidase activity increases in senescent cell up to a degree that surpasses a threshold level that renders the activity detectable at a suboptimal pH 6.0 [21,22]. A later study clearly demonstrated that the SABG activity arises from the lysosomal β-galactosidase 1 (GLB1) gene product [23]. In senescence cells, both the mRNA and the protein levels of this gene are significantly elevated, and the enzymatic activity increases concomitantly [23]. Furthermore, the enhanced enzymatic activity in senescence can be measured both at the optimal pH for activity - pH 4.5 as well as at the suboptimal pH 6.0. These findings demonstrate that the significantly increased SABG activity at senescence is the basis for the activity detected at the suboptimal pH 6.0, and therefore used as a marker for senescence [23].

The extent of the senescence-induced increase in lysosomal β-galactosidase can be measured by Western blotting or soluble enzymatic activity [23], however the activity of β-galactosidase is most easily and robustly detected by histochemical staining with X-gal serving as a substrate [23]. In addition to age related accumulation of lysosomal β-galactosidase in senescent cells probably due to the increased lysosomal content in the cell, other yet unknown factors such as functional differences in senescent lysosomes may contribute to the very high levels of β-galactosidase observed by SABG staining at pH 6.0 [23].

Despite its ease and utility, concerns have arisen regarding the specificity and reproducibility of the SABG assay. Studies that question the specificity of the SABG assay as a CS marker have found SABG activity in quiescent cell cultures [24], confluent non-transformed fibroblasts cultures [25,26] and in serum starved cells [26]. Other concerns relate to the nature of the semi-quantitative measure. Previously described scoring has been based on experimentalist-dependent determination of a given cell as being positively or negatively stained, and may not be anchored in reproducible criteria. Furthermore, the intensity of blue staining in positive cells may be difficult to quantify, such that cells with strong, moderate, or weak blue staining may all be recognized as equally positive. This renders the method insensitive to subtle effects of various stressors on CS, and might contribute to the inconsistency in replicating SABG assay results in skin biopsies [25].

The foregoing motivated us to develop a quantitative *in situ *SABG assay, which could be more easily applicable and reproducible in the study of CS both *in vitro *and *in situ*. We have utilized the framework of the widely utilized protocol of the *in situ *SABG assay [16], and applied digital-image processing in order to perform quantification of the assay staining. In addition, we have also varied the pH of the assay to broaden the range of histochemically detectable activity. We show validation of this quantitative *in situ *SABG assay on cultured human foreskin fibroblasts and frozen kidney biopsies from normal and diabetic mice, stained under different assay conditions. The values derived from this analysis are termed β-galactosidase activity values (BGAVs) and are highly sensitive and reproducible.

## Results

### Image processing of the standard SABG assay

**Figure 1:**
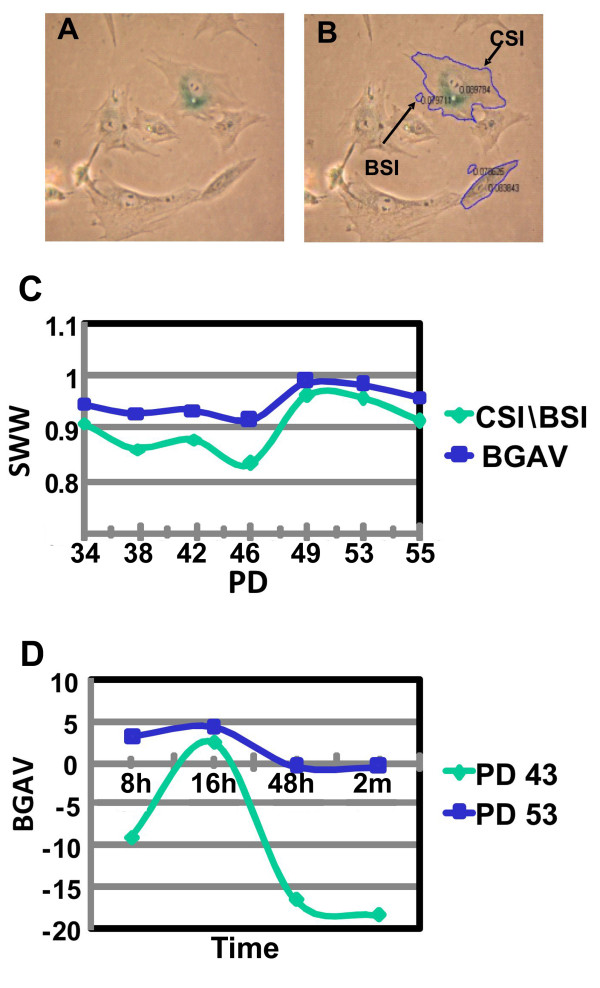
**Application of a digital image-processing tool to the standard SABG assay**. **A**. SABG staining of FSE cells (PD 34) at pH 6.0. **B**. Image analysis of the field shown in Figure 1A using the Matlab tool described in "Materials". The cell membrane borders and the background control area (small circles) were marked manually. The logarithmic ratio of the cytoplasmic staining intensity (CSI) divided by the background staining intensity (BSI), yields the BGAV. **C**. Shapiro-Wilk W (SWW) normality test applied to the CSI/BSI values (green line) and to the BGAVs (blue line) obtained from FSE cells at progressively increasing PDs. For any given PD of the FSE cells, the SWW normality value of the logarithmic ratio (BGAV) was higher than the CSI/BSI ratio, indicating a better fit with a normal distribution when applying logarithmic transformation to the CSI/BSI ratio. **D**. BGAV variation over time. Measurement of BGAVs of FSE cells at different time periods after completion of staining yielded a parabolic distribution of the BGAVs, peaking at 16 hours, and reaching a plateau after 48 hours which remains as such for as long as 2 months following the completion of the staining reaction. This analysis was carried out for FSE cells at two different PDs: PD 43 (green line) and PD 53 (blue line). Hour (h), month (m).

The SABG assay produces blue-green staining in the cytoplasm of positive cells, albeit there is great variability in the degree of staining among the positive cells. In order to objectively quantify this staining we utilized the protocol for SABG staining as previously described [16], and proceeded to capture and digitally analyze images of the stained cells. This quantification method, based on color intensity analysis of the photographed images, generates values of Cell Staining Intensity (CSI) measured in arbitrary intensity units. CSI values are the product of the ratio between the green plus blue values of each pixel in the manually marked cell cytoplasm and the total color (red, green and blue) in the same cytoplasm, divided by the cell surface area. Standardization of each single CSI value is achieved by dividing the CSI value with the adjacent background staining intensity (BSI) value (Figure 1A, B). We found that the background is uneven over the photographed area and therefore standardization to the figure background is best achieved by marking a background area adjacent to each analyzed cell. In order to decrease the signal to noise ratio, we applied a logarithmic transformation to the ratio CSI/BSI and termed the value obtained: BGAV. For each experiment a minimum of 250 cells were analyzed to generate BGAVs, which were further analyzed as mean values and distributions.

To apply this analysis to primary human fibroblasts aged in culture and in order to determine the preferred analysis strategy, we stained and calculated CSI/BSI and BGAVs for two different primary fibroblast cell lines, FSE and BJ, at various population doublings (PDs). We applied normality tests for these values, and found higher normal distribution indices for BGAVs versus CSI/BSI (Figure 1C). The Shapiro-Wilk W (SWW) BGAVs of 250 FSE cells at PD 38 was higher than the CSI/BSI ratio of the same cells, yielding a better fit to a normal distribution. Nevertheless, in most instances the BGAVs did not fit a normal distribution according to either Shapiro-Wilk W or Kolmogorov-Smirnov normality tests, and therefore in our statistical analysis we used the Kolmogorov Smirnov test, rather than the Student's t-test, which assumes normal distribution.

The first scoring of BGAVs was performed on images captured 8 hours following the start of the incubation with the X-Gal staining solution, immediately after stopping the staining reaction by washings with PBS, as described in "Methods". In order to examine whether the BGAVs change as a function of the time elapsed since the staining was completed, we continued to measure the mean BGAV of 250 cells at different time points following staining. We observed a parabolic behavior of the mean BGAV over time, peaking at 16 hours after the termination of the staining reaction (Figure 1D). The BGAVs reached a plateau 40 hours after the staining reaction was terminated and remained stable for as long as two months. We have chosen the values attained at the plateau stage as more reliable and stable markers for quantifying the degree of SABG staining and proceeded to acquire all our images at least 40 hours after completion of staining.

### BGAVs in Cellular Senescence pathways

**Figure 2:**
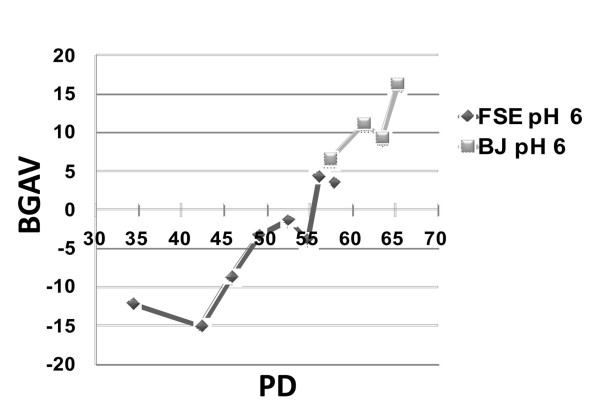
**BGAVs change with increasing population doublings**. The BGAVs obtained at pH 6.0 at progressively increasing PDs of both FSE and BJ fibroblast cells. FSE cells were analyzed between PD 34-58 and BJ cells were analyzed between PD 57-65. Both cell types were stained for SABG activity at weekly intervals.

The SABG assay was originally introduced to measure RS [16]. In order to confirm that our quantification analysis indeed detects this type of senescence, we measured the mean BGAV in FSE and BJ fibroblasts at progressively increasing PDs. Our measurements in the FSE cells demonstrated a steady increase in BGAVs between PDs 34-58, and in BJ fibroblasts between PDs 57-65 (Figure 2). Analysis of cell surface areas from FSE cells at PDs 34 to 56 presented a concomitant gradual increase in the mean cell surface area from 24520 pixels to 39125 pixels respectively as expected in cells approaching CS, and this change was statistically significant (p < 10^-5^). We fully appreciate the fact that accurate cell area can only be calculated using confocal microscopy, though for the purpose of validating the quantitative SABG assay we did not consider this to be necessary. The increase in mean cell surface area would be expected to yield a lower mean BGAV in cells approaching CS, since cell surface is the denominator for CSI. However, as demonstrated in Figure 2, the increase in the mean BGAV from -12.2 ± 13.7 at PD 34 to 4.3 ± 14.7 at PD 56 (p < 10^-5^), validates the efficacy and reliability of this method to observe and measure changes in SABG staining associated with RS.

**Figure 3:**
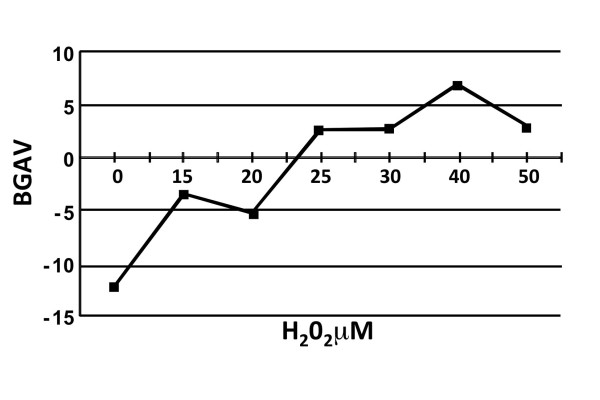
**H_2_O_2 _Stress-Induced Premature Senescence measured by BGAVs**. BGAVs of FSE cells stained at pH 6.0 increased progressively with exposure to increasing concentrations of H_2_O_2_. Concentrations of H_2_O_2 _higher than 50 μM resulted in cell death such that the cells could not be analyzed for BGAVs.

Many studies have shown that SIPS is also associated with positive SABG staining [12,27]. In order to further validate the reliability of our method, we exposed FSE cells at PD 34 to gradually increasing concentrations of H_2_O_2 _in order to induce acute SIPS. Indeed, an H_2_O_2 _concentration dependent increase in the mean BGAV was observed (Figure 3). The mean BGAV was significantly higher after exposure to 15 uM H_2_O_2 _(-3.4 ± 17.8) in comparison to no added H_2_O_2 _(-12.2 ± 13.7) (p < 10^-5^). No significant differences in BGAVs were observed between H_2_O_2 _concentrations of 15 uM and 20 uM (p = 0.2183). BGAVs reached a plateau at 25 uM H_2_O_2_. Cell death occurred when cells were exposed to H_2_O_2 _concentrations higher than 50 uM such that the cells could not be analyzed for BGAVs at these concentrations. Hence, BGAVs appear useful in documenting SIPS-induced SABG staining, as well.

### The effect of pH on BGAVs

**Figure 4:**
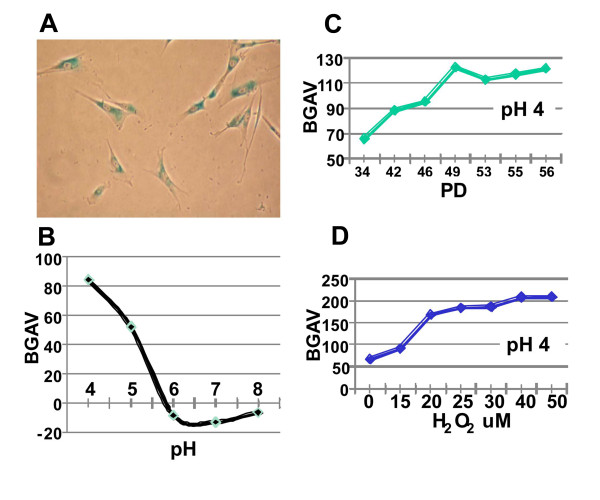
**pH effect on BGAVs**. **A**. FSE cells at PD 34 stained at pH 4.0. All cells demonstrated some degree of staining. **B**. BGAVs are inversely related to the pH at which the reaction was carried out. Staining was performed on FSE cells at PD 42 under different pH conditions. **C**. The effect of increasing PDs on BGAVs obtained at pH 4.0. FSE cells at progressively increasing PDs were stained at pH 4.0 and analyzed for BGAVs. **D**. The effect of H_2_O_2 _on BGAVs obtained at pH 4.0. FSE cells were treated with progressively increasing H_2_O_2 _concentrations and BGAVs were determined at each concentration.

Recent studies have demonstrated that SABG activity at pH 6.0 in mammalian cells emanates from the activity of the lysosomal enzyme GLB1 [23]. This pH is suboptimal for this enzyme's activity that normally is carried out in the highly acidified lysosomes. The optimal pH for lysosomal β-galactosidase activity in human fibroblasts and leukocytes is situated between pH 4.2 - 4.6, and activity declines by approximately 25% at pH 5.0 and by 50% at pH 5.5 [28]. Apparently, only senescent cells with high β-galactosidase activity yield detectable staining at pH 6.0 [23]. Accordingly, it has been the accepted convention to date to carry out the SABG assay at pH 6.0, since at pH 4.0 most cells are positively stained due to basal lysosomal β galactosidase activity (Figure 4A) and therefore differentiation between levels of activity was considered not feasible at pH 4.0. However the β-galactosidase activity measured in late- compared to early-passage fibroblasts was six to seven-fold higher both at pH 4.5 and pH 6.0 [23]. We therefore proceeded to study whether our quantification method might be able to measure differences in staining intensities obtained at pH values other than pH 6.0 in fibroblasts at early and late PDs. To this end, we first measured BGAVs obtained at different levels of pH in FSE cells at PD 39, a stage at which fibroblast cells are barely stained at pH 6.0. As shown in Figure 4B, the BGAVs decreased as the pH increased, and the highest BGAVs were attained when staining was performed at pH 4.0. Following this finding we analyzed the BGAVs obtained at pH 4.0 during RS and SIPS. We observed that both at RS (Figure 4C) and SIPS (Figure 4D) staining at pH 4.0 increased the resolution between the BGAVs at low PDs, or at low H_2_O_2 _values. While the mean pH 4.0-BGAV at PD 34 (65.6 ± 45.5), was significantly lower than at PD 42 (88.4 ± 60.4) (p = 0.0001) (Figure 4C), this significant difference could not be documented at the same PDs with pH 6.0-staining (Figure 2). Similarly, readily discernible differences were obtained while comparing the mean BGAV at 15 uM H_2_O_2 _compared with 20 uM H_2_O_2 _when staining was done at pH 4.0, however this was not the case at pH 6.0. Therefore, we conclude that in order to measure smaller increments in the SABG activity or to perform this assay more accurately, it is beneficial to perform the staining procedure also at pH 4.0 due to the increased sensitivity of the BGAVs at this pH. However this requires a rigorous quantitative assay for histochemical staining intensity in order to distinguish the range of intensity of positive staining.

### The effect of cell density on BGAVs

**Figure 5:**
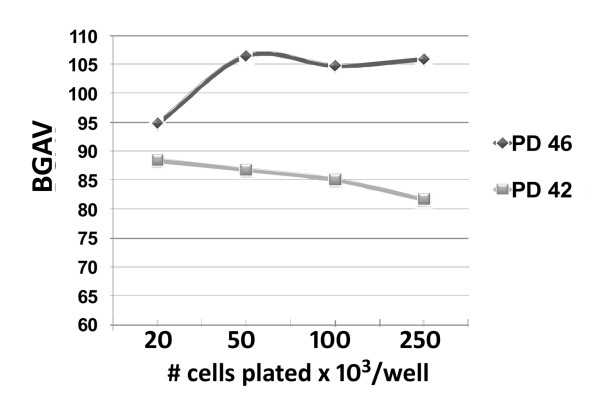
**Cell Density and BGAVs**. FSE cells at PDs 42 and 46 were plated at increasing densities (20,000 -250,000 cells/well) and stained at pH 4.0. BGAVs were determined at the different cell densities.

Earlier studies have reported that the degree of SABG staining is influenced by cell density, such that more intense staining is obtained when cells are closer to confluence [25]. In order to determine whether confluence effects are evident using our analytic approach, the mean BGAV was determined in wells at different confluence levels. ANOVA analysis of the mean BGAV at pH 4.0 in two duplicate experiments using FSE cells showed no differences with varying cell density (p = 0.1), even when cells were plated at a high density (250,000 cells per well) (Figure 5).

### *In situ *SABG assay

**Figure 6:**
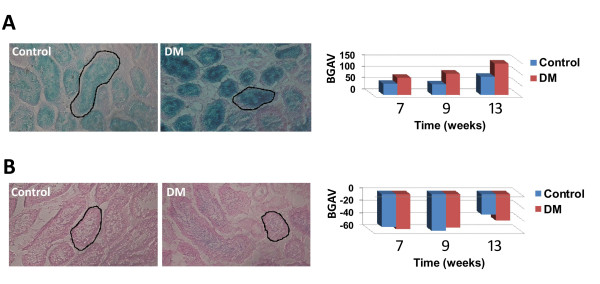
***In situ *kidney SABG activity in diabetic and non-diabetic mice**. SABG staining was carried out for kidney sections, and renal tubule-BGAVs were measured for both control and diabetic (DM) mice at 3 time points (7, 9 and 13 weeks) at pH 4.0 (**A**) or pH 6.0 (**B**). DM mice were analyzed by two independent experiments at the 9- and 13-week time points for each pH. Cross-sections are from kidneys at the 13-week time point, for both control and diabetic mice. **A**. At all three time points SABG staining was apparent and BGAVs for the diabetic mouse was significantly higher than those for the control mouse (7 weeks p < 0.004; 9 weeks p < 0.0009; 13 weeks p < 10E^-8^). Values at the 9- and 13- week time points for the DM mice represent the mean of two independent experiments (SD = 35.9 and 27, respectively). A black line in both tissue samples demarcates a positively stained tubule. **B**. No SABG staining was apparent at pH 6.0 for control and diabetic mice and renal tubule-BGAVs were not significantly different between the groups. Values at the 9- and 13- week time points for DM mice represent the mean of two independent experiments (SD = 4.6 and 18.6, respectively). A black line in both tissue samples demarcates a negatively stained tubule.

The potential roles CS plays in organismal aging and in tumor suppression raise the importance of a reliable quantitative method to determine CS *in vivo*. We have chosen to focus in the current study on kidney biopsies, in which CS has been described in various forms of kidney pathology [29-31]. Specifically, we have focused on type 2 diabetes mellitus (DM) wherein increased CS, quantified by SABG and p16INK4a, has been demonstrated in human subjects [32]. We have used a well-accepted streptozotocin (STZ) model of murine diabetes mellitus [33] in order to analyze SABG activity in renal tubules 7, 9 and 13 weeks following the administration of STZ. At pH 6.0 no positively stained kidney cells were detected and analysis by our software indicated that the mean BGAV was not statistically different between diabetics and controls at all 3 time points (Figure 6B). We hypothesized that β-galactosidase activity in mouse kidney cells may be low and detectable only at the optimal pH, and therefore applied the SABG assay also at pH 4.0. Indeed, the BGAVs measured in tubular cells at pH 4.0 differed significantly between the kidney samples of the diabetic mouse and the controls at all three time points (7 weeks p < 0.004; 9 weeks p < 0.0009; 13 weeks p < 10E^-8^) (Figure 6A). The staining was specific to tubular cells and no positive staining was detected in the glomeruli or interstitial tissue from both diabetic and control mice. In addition, the analysis revealed that the BGAVs at pH 4.0 increased gradually in both diabetics and controls between week 7 and week 13 (p = 5E^-6^, 6E^-6 ^respectively) reflecting changes in SABG activity most likely due to aging.

**Figure 7:**
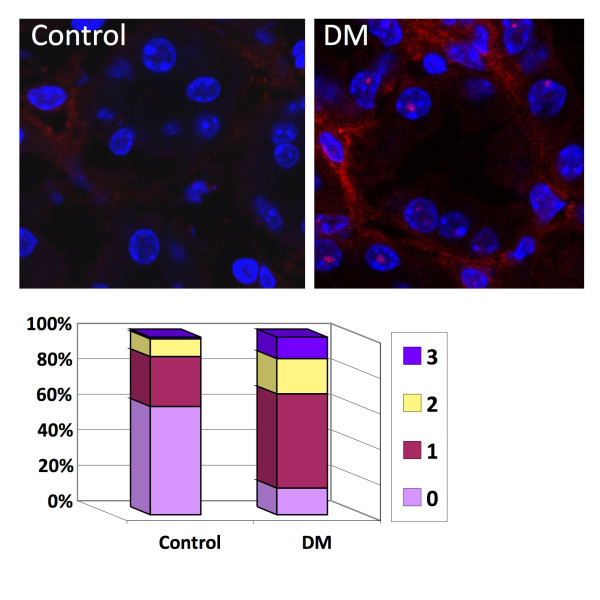
**γ-H2AX foci in diabetic and non-diabetic mice**. γ-H2AX staining was carried out for kidney sections at the 13-week time point for both control and diabetic (DM) mice. Red - γ-H2AX signals, Blue - DAPI nuclear staining. One hundred tubule cells from each sample were analyzed for the number of γ-H2AX foci present in the nucleus. The percentage of cells displaying 0-3 foci per nucleus appears in the graph (3 foci was the maximum number of foci detected per nucleus).

In order to verify that the SABG staining indeed detects senescent cells in the kidney, we proceeded to analyze the kidney samples for an additional marker of senescence. We performed immunofluorescent (IF) staining with an antibody for γ-H2AX, a quantitative indicator of senescence both *in vivo *and *in vitro *[34]. One hundred nuclei in tubular cells of both diabetic and control mice at the 13-week time point were scored for the number of foci present in the nucleus (Figure 7). The number of nuclei containing one or more γ-H2AX foci was significantly higher in the tissue originating from the diabetic mouse in comparison to the control mouse (Pearson's chi-square = 44.9, p = 2.1E^-11^). Moreover, by comparing the percentage of γ-H2AX-positive nuclei with pH 4-SABG-positive tubules (defined as demonstrating BGAV values greater than the average score for the corresponding control tubules), the correlation coefficient was calculated to be 0.96, providing further reassurance that indeed the SABG staining at pH 4.0 identifies senescent cells.

## Discussion

Cellular senescence is a final common pathway for a number of DDR pathways, and appears to play a major role in normal aging, acute stress conditions, and possibly as a tumor suppressor mechanism [5,14]. Despite its wide relevance to both normal tissue kinetics, and organismal aging, CS lacks definitive well-characterized and easily measured biomarkers. Senescence-associated β-galactosidase staining has been the most widely used technique to characterize senescent cells, however several recent studies highlight a number of caveats using this assay [25,26,35]. A major limitation of the SABG assay is the subjective assignment of cells as being positively or negatively stained. As a result, new quantitative SABG assays have been developed. These methods include measurement in cell extracts of the conversion rate of 4-methylumbelliferyl-D-galactopyranoside (MUG) to the fluorescent hydrolysis product 4-methylumbelliferone (4-MU) [36] and a method based on the levels of fluorescein di-(β)-D-galactopyranoside in the suspension buffer [37]. A quantitative chemiluminescent assay has also been recently reported [38], as well as FACS staining of SABG [39]. All these methods have yielded promising results for a quantitative measure of SABG, but are not designed for *in situ *quantification of SABG activity. We have used the platform of the original SABG assay [16] as the basis for developing an *in situ *reproducible quantitative assay. The digital image-analysis we describe herein enables the quantification of SABG staining in uniform units, and distinguishes different degrees of staining. In addition, our analysis suggests that SABG staining is not influenced by cell density up to a certain degree of confluence in either pre-senescent or senescent cells (Figure 5).

A further modification and validation of this assay is varying the pH at which the assay is carried out to include pH 4.0, at which biochemical activity of the GLB1 product is significantly higher [23]. The original description of SABG assay at pH 6.0 was meant to generate a threshold at which only cells with senescence-dependent accumulation of the enzyme (now known to be the GLB1 gene product) and consequently higher activity stain positively. Qualitative SABG assay at a higher pH might be simpler and faster for interpretation when striking qualitative differences are being assessed. However, staining at pH 4.0 renders the assay more sensitive to subtle changes, such as occur under conditions of metabolic stress, as demonstrated by the intensity of the staining after treatment with hydrogen peroxide. SABG staining at pH 4.0 also enabled identification of CS in kidney samples of diabetic mice that was not evident at pH 6.0. While pH 4.0-4.5 is the optimal pH for SABG activity, [36,40], without the digital analysis, subjective scoring will lose its utility for determination of CS as most cells will seem to be positively stained. Thus, in conclusion the pH of the assay should be adjusted in accordance with the biological question.

An evident advantage of a histochemical assay over a fluorimetric approach, as described by Yang and Hu [37], is the capacity for *in situ *quantification to shed light on CS at the level of individual cells and not only at the level of a whole cell population. Furthermore, the main innovation in our method is the ability to quantify *in situ *mixed cellular tissue sections and evaluate the SABG activity in specific cell types. Since many tissues are constituted of various types of cells, only the *in situ *staining can provide insights into the type of cells in a mixed population demonstrating high SABG activity.

As noted above, *in situ *visualization of SABG in diabetic mouse kidneys was possible only when the SABG assay was performed at pH 4.0. Previous SABG activity studies on *in situ *samples [16] have shown a qualitative increase in SABG activity in tissues from elderly patients even at pH 6.0. However no such staining was evident at pH 6.0 in the kidneys of the diabetic mice in the current study. Other studies in which SABG assay was used as a marker for CS in tissues have also frequently yielded conflicting results [25], and it has been suggested that activity is not causally related to CS induction [23]. In this study we have confirmed by an independent assay that indeed the kidneys of the diabetic mice harbor senescent cells. Herein we suggest that the ambiguity related to the SABG staining as a CS marker in tissues might have been the result of measurement of SABG activity at a high and suboptimal pH. However, once again we emphasize that extending the assay to the more sensitive pH of 4.0 must be coupled with quantification of staining to achieve interpretable results.

## Conclusions

Utilization of an objective quantitative measurement of SABG activity by digital image analysis has proved to be accurate and reproducible in detecting cellular senescence derived by several stimuli. The increased SABG activity observed among *in situ *tissue sections from diabetic mouse kidneys was observed only when the assay was performed at a physiologic pH 4.0, and the analysis was enabled due to the use of computer aided image analysis. A major advantage and novelty of this approach in comparison to other qualitative and quantitative SABG methods is the ability to analyze tissue sections in the study of *in situ *senescence.

## Methods

### Cell culture conditions

Human primary foreskin fibroblasts (FSE) [41] and BJ [42] (obtained from Woodring Wright, University of Texas, Southwestern Medical Center, TX, USA) were cultured and brought to replicative senescence as described previously [41].

### H_2_0_2 _treatment of cells

FSE cells were exposed to H_2_O_2, _diluted in PBS, at different concentrations (0-100 μM). Following incubation with H_2_O_2 _cells were washed with PBS and grown for an additional 48 hours in culture medium prior to staining. Control cultures at the same population doubling followed the same schedule of medium changes without exposure to exogenous H_2_O_2_.

### Senescence associated β-galactosidase assay

Cells were seeded 48 hours prior to staining at 2-4 × 10^4 ^cells/well in six well plates. This cell density ensures that the staining is performed before the cultures reach confluence. SA-β-Gal staining was performed as previously described with minor modifications [16]. Briefly, the cells were washed with cold PBS, and fixed for 5 min with 0.5% glutaraldehyde diluted in cold PBS. After fixation, cells were washed in PBS and incubated for 8 hours at 37°C in staining solution containing 1 mg/ml 5-bromo-4-chloro-3-indolyl-β-D-galactoside (X-Gal) (Roche) and the rest of the components described in [16]. For staining at different pH values, 0.1 M citric acid and 0.2 M Na_2_HPO_4 _solutions were mixed at appropriate proportions. For pH 4.0 - 38.6 ml of 0.2 M Na_2_HPO_4 _were mixed with 61.5 ml of 0.1 M citric acid, and for pH 6.0 - 63.2 ml of 0.2 M Na_2_HPO_4 _were mixed with 36.9 ml of 0.1 M citric acid. Following the incubation period at 37°C, cells were washed 3 × 5 minutes with cold PBS and stored in PBS at 4°C until images were collected.

For analysis of the effect of cell density on SABG activity, FSE cells from two different PDs (42 and 46) were seeded at increasing densities ranging from 20 × 10^3^/well to 250 × 10^3^/well (in 6-well plates), and cultured until the well containing the highest cell density reached confluence.

### Diabetic animal model

Two-month-old C57Bl/6 mice were injected intra-peritoneal for 5 consecutive days with the pancreatic islet β cell toxin, STZ, at a concentration of 50 mg/kg dissolved in 50 mM citrate buffer pH 4.5. Blood glucose levels were monitored once a week with DM being defined as a consistent blood glucose level higher than 200 mg/dl. Mice were sacrificed at 3 time points (7 weeks after injection, 9 weeks after injection and 13 weeks after injection). The experiment was repeated twice for the 9- and 13-week time points. Anesthesia was carried out by injection of a mixture of ketamine and xylazine. Kidneys were removed and embedded in optimal cutting temperature compound (Tissue-Tek, Sakura), snap-frozen in liquid nitrogen-cooled methylbutane and stored at -70°C. The institutional animal care and welfare committee approved these experimental protocols.

### *In-Situ *SABG staining

Frozen kidney sections were cut at 6 μm and kept on dry ice until further processed. Slides were fixed with 2% formaldehyde/0.2% glutaraldehyde in PBS for 5 minutes. After washing with ice cold PBS, slides were incubated over night at 37°C with SA-β-Gal staining solution previously described [16]. Following staining, slides were incubated for 48 h in ice cold PBS, and then were counterstained with eosin, dehydrated, and mounted as described [16].

### Microscopy, image analysis and BGAV analysis

#### Analysis of tissue-culture cells

Color images of SABG stained tissue culture cells were captured on a Nikon eclipse TS100 inverted microscope using a 10× objective and a Nikon Coolpix E995 digital camera 40-48 hours post staining. In several cases, specific wells were photographed several times at different intervals after staining was completed (8 hours to 2 months) to determine whether BGAVs change over time.

Quantitative analysis of the images was performed using a Matlab application for cell marking (SegmentGui) and color analysis (detailed instructions and download appear on website http://md.technion.ac.il/pictures/storage/45/47.zip). A minimum of 250 randomly chosen cells was marked manually for each measurement point.

#### Analysis of stained tissues

The images of stained tissues were captured on a BX51 Olympus microscope using a 20× objective with an Olympus DP70 digital camera controlled by analySIS software (Soft Imaging System). Image acquisition was performed with the following fixed color settings: red = 0.36, green = 0.92, blue = 0.85, offset = 504, and exposure time of 6.8 ms.

Analysis of BGAVs was performed on whole renal tubules (Figure 6). At least 50 tubules were analyzed from each mouse (DM and control) at each of the 3 time points. Experiments were carried out in duplicates for the DM mice at the 9- and 13-week time points.

### Statistical Analysis

The distributions of SABG values were compared to a normal distribution by the use of Shapiro Wilk W normality test. Since most distributions deviated significantly from a normal distribution (see "Results"), we used the two sample Kolmogorov Smirnov test to calculate distribution differences between the different cell samples and for calculation of the p values.

### Immunofluorescence analysis of γ-H2AX foci

The staining of the mouse kidney tissue with antibodies for γ-H2AX was carried out as described previously [2]. Briefly, 8 μM sections were cut from the frozen kidney biopsies and immediately fixed for 20 minutes in freshly prepared 4% paraformaldehyde diluted in PBS. Following the fixation, the tissue sections were permeabilized for 20 minutes with PBST (PBS + 0.2% Triton X100). Sections were blocked for 1 hour in blocking solution consisting of PBS + 4% BSA at room temperature (RT) and incubated over night at 4°C with anti-γ-H2AX (S139, Upstate; Chicago, IL) diluted 1:750 in blocking solution. The following day, the slides were washed 3 × 5 minutes in PBST at RT, and incubated with a secondary antibody (Cy3- conjugated donkey anti-rabbit antibody) for 1 hour at RT, Slides were then washed 3 × 5 minutes in PBST at RT and mounted with Vectashield containing DAPI (H-1200, VECTOR LABORATORIES). Fluorescence signals in stained tissue were analyzed using laser scanning confocal microscopy (LSM 510 Meta Confocal, Carl Zeiss Inc. Germany). Nuclei were scored for the number of γ-H2AX foci present in each nucleus. Differences in number of foci between diabetic and control tissues were compared by Pearson's chi-square test.

## Authors' contributions

LIS conceived the study, participated in design of the study, carried out experiments, analyzed data, and participated in writing the manuscript. SI and AC developed the software. AR, RB, SY and HS carried out the experiments and participated in analysis of the data. SS and KS conceived the study, participated in design of the study, and wrote the manuscript. All authors read and approved the final manuscript.
